# The Relations of Medical Men to Each Other, and the Public

**Published:** 1864-10

**Authors:** 


### EDITORIAL DEPARTMENT.

### THE RELATIONS OF MEDICAL MEN TO EACH OTHER, AND THE PUBLIC.

This is a theme of vast scope and interminable length; one which has often received the most mature thought and been portrayed in the plainest and most convincing terms; though the relations and duties of physicians to each other and the public have not yet been sufficiently understood and appreciated, to guide the conduct and rightly influence the motive of some, even of those who are reverenced as the Fathers in Medicine. The old adage of "who shall decide when doctors disagree," was applicable only to the early differences of opinion which prevailed upon the vexed questions in pathology, and cannot be supposed to indicate that physicians had any personal differences which could not be decided. It only forcibly expresses the great truth, that the causes and location of diseases, whether in the solids or fluids, about which there were differences of sentiment, could not be decided in the state of medical knowledge which obtained at that period; for no one is willing to suppose that the early fathers of medicine did not entertain for each other the highest respect, and treat each other with the greatest politeness. The public however have placed a widely different construction upon this old sentiment, which only showed activity of thought and ambition to excel, and was in no way indicative of professional disagreement. If one physician thinks all disease located in the blood, and another that all diseases are primarily in the nerves, they have no differences which it is of any great importance to decide, provided in treatment they agree. It is, however, sometimes seen that men construe differences of opinion and different views of evidence, into personal opposition, and fancy that those who do not wholly adopt their views even in matters of scientific inquiry, are opposed to them in feeling, and do not regard their welfare or desire to promote their success. The bonds of union and good fellowship in the profession are really very strong, and there is no organization, secular or religious, in which the duty "to love one another" is more binding than in the medical profession; physicians are united by the ties of a common interest, and they should have at heart the common good. The motive for professional "good manners" is very great, and a departure from the rule of "doing by others as you would have them do by you," in all matters of professional intercourse is sure to bring ultimate disgrace. There is no honor or fame in medicine which is not dependent upon the favor of physicians, and whoever attempts to gain the higher rewards of professional success, must never forget to regard with zealous care, the feelings and interests of his brother associates. From them he will receive his true merits, and the estimation which they place upon him, will finally become his market value with the public.

Physicians are perhaps brought most intimately in contact with each other in consultations, and it is here that good breeding is most observable. The American Medical Association has established some rules in consultations which it would be better if they were more carefully observed, but it is no part of our design to suggest any new regulations or urge especial attention to the old ones already adopted; these rules should however be well understood. Consultations are sometimes held strictly under these regulations, and are yet very distant from the true standard of professional good faith. and on the other hand they are not always to be strictly followed, since the consulting physician who desires solely the best good of the patient, and seeks to promote the interests of the medical attendant before his own, may in some things take his own way to accomplish such object. Consultations are to be favored in difficult and dangerous cases, for the promotion of medical science by comparison of views, also for satisfaction of friends, but especially to increase confidence in the medical attednant; while in no instance is there any occasion for consultations for the purpose of impressing the patient or any of the friends with the importance or attainments of the consulting physician.

Consultations are mainly to determine the nature of obscure diseases, and not to introduce the special forms of medication which a consulting physician may have found useful, in the place of those of the regular attendant. They are to strengthen, approve and confirm the course which has been adopted, while to suggest a change of medication because patient or friends look eagerly for "something more to be done," when in truth all proper means have been adopted, is one of the most disgraceful propositions ever suggested by a consulting physician. It would seem that such a thing could never have been conceived of, but it is repeatedly urged upon medical attendants upon the ground that "something more will be expected" by the friends, since they have had the expense and trouble of calling another physician. It is clearly the duty of physicians to do all in their power for the relief and cure of disease, and this obligation is always binding whether in attendance or consultation; but variety of medication or increased experiment with remedies, is really no part of the object of consultation. The nature of the disease, its natural course, and its probable termination are prime subjects for consideration, and the view that a consultation is called to decide what remedies or combination of remedies shall be administered is founded largely in ignorance and misapprehension. If invited by the attendant, it is proper for the consulting physician to suggest any changes he may please in the treatment, that is, if he believes any important variation in the general plan would be useful; but to urge, to change of remedies, essentially the same in their nature as those already adopted, or to suggest new and comparatively untried remedies, because they are new and untried, is unprofessional and disgusting in the extreme.

Consultations are often called in confirmed disease, which, from its very nature, is necessarily fatal; this is often desirable in order to divide the responsibility of deciding such cases, and for the gratification of patient and friends. The friends often desire to hear combined expression of opinion, upon some, or all, of the various points of interest. A plain statement of opinion should not be withheld, and it is not improper for consulting physician to express his opinions, especially those in known harmony with the views of the attendant. It is his duty to inform them plainly, that medicine in such disease is not curative, merely paliative, if indeed at all useful, and that it is vain to look farther than to their family attendant for advice. Patients look too eagerly for medicine, and with them, some times, the more you will give, the more you are worth. Consultations are for their good, oftener that they be satisfied with less, rather than served with more. I remark npon this point because in my life I have heard a professional brother after consultation remark to a family whose attendant had mostly omitted medication, "I would give tinc—— and solution of ——. We do not like to fold our arms and let our children die, without trying something, even if we are unacquainted with any medicine which is curative of such disease." That such a narrow-minded. impolite, ignorant, and ungentlemanly physician should ever have been called in consultation is remarkable. He had no professional reputation and was called through the influence of a neighboring nurse. Professional good manners do not depend upon rules and medical codes; they are the natural product of a generous and noble soul. Rivalry and competition are inadequate under any circumstances to degrade the conduct of a competent, well educated physician, and no amount of medical legislation can breathe a noble and generous impulse, into a mean and selfish spirit.

There is sometimes complaint that one physician has received and treated another's patient; and upon this point we have a word to offer, though we fear our views may be unlike some in the profession. If from any cause, a change of physicians is desired, it is clearly right that such wish be gratified, and when a physician in attendance has been notified of a desire and design to change, whoever succeeds him, has no physician's patient but his own. People who have for years called one physician, are always at liberty to call another if they choose; and there is no ground of complaint by either the refused or chosen, unless it mayhap, bills are unpaid, in which case the dismissed physician wins, and the called, loses. There are quite a large class of families, who fancy to invite the greatest possible variety of medical attendants, and are never satisfied long with the same man; when they leave, you win; when such invite your attendance, you lose. *Interference* with another physician's patient, is too mean to be thought of; but whoever consults you, or calls you, and follows your advice, is your patient; when he consults another, and follows his directions, he is his patient, and your claim has expired. We believe in the greatest freedom in all this matter of selecting medical advice, and have no sympathy with the notion that families or individuals are not always at liberty to ask such medical attendance as they choose. It is better for families to have their regular medical attendant, and to adhere to him; but they are nevertheless at perfect liberty to change.

There is one class of families who rarely call the same physician the second time; they pay their bills in this sort of *change*. We have often thought of suggesting the registration of their names for a list, from which any might choose patients who desire them.

One other point in the relations of physicians to each other and the public and we close our rather long drawn out editorial. Uniformity in the prices for professional services is much to be desired; "upon this rock we split." We do not propose to say that professional services are uniformly of the same value, but there should be some fixed standard below which it should be considered unprofessional and disgraceful to render charge. In our city, some of the oldest and most thoroughly established physicians, render their services to the wealthiest citizens, at prices which would beggar a young practitioner who depended upon his professional income for support. It is not believed that they are the more valued for the low price at which they hold themselves; they do however sometimes in this way retain the business of penurious misers who have grown comparatively rich by immense contraction of themselves; but these physicians have never grown to distinguished eminence, and their services are generally estimated at about their own standard. It is the duty of physicians to guard and protect each other in this matter as in all other matters of professional interest and honor. The fee bill as established should be regarded; it is a shame and disgrace that its spirit and letter are so often violated. We are not however without one consolation; those who prize most highly their services, are generally regarded as of highest value; while the cheap physician is never the most respected.

As a profession we should cultivate the purest motives, and be actuated by self-sacrificing devotion for the common good; as individuals we should remember that all our noblest objects of professional ambition, all the higher rewards of success, are obtainable only through an estimate of our merits, by the members of an honorable profession.

